# Improving dengue fever predictions in Taiwan based on feature selection and random forests

**DOI:** 10.1186/s12879-024-09220-4

**Published:** 2024-03-20

**Authors:** Chao-Yang Kuo, Wei-Wen Yang, Emily Chia-Yu Su

**Affiliations:** 1 Smart Healthcare Interdisciplinary College, National Taipei University of Nursing and Health Sciences, No.365, Mingde Road, Beitou District, Taipei City, 112303 Taiwan; 2 Graduate Institute of Biomedical Informatics, College of Medical Science and Technology, Taipei Medical University, No.301, Yuantong Road, Zhonghe District, New Taipei City, 23564 Taiwan; 3 Clinical Big Data Research Center, Taipei Medical University Hospital, No.252 Wuxing Street, Xinyi District, Taipei City, 110 Taiwan

**Keywords:** Dengue fever, Machine learning, Air quality index, Random forests, Feature selection

## Abstract

**Background:**

Dengue fever is a well-studied vector-borne disease in tropical and subtropical areas of the world. Several methods for predicting the occurrence of dengue fever in Taiwan have been proposed. However, to the best of our knowledge, no study has investigated the relationship between air quality indices (AQIs) and dengue fever in Taiwan.

**Results:**

This study aimed to develop a dengue fever prediction model in which meteorological factors, a vector index, and AQIs were incorporated into different machine learning algorithms. A total of 805 meteorological records from 2013 to 2015 were collected from government open-source data after preprocessing. In addition to well-known dengue-related factors, we investigated the effects of novel variables, including particulate matter with an aerodynamic diameter < 10 µm (PM_10_), PM_2.5_, and an ultraviolet index, for predicting dengue fever occurrence. The collected dataset was randomly divided into an 80% training set and a 20% test set. The experimental results showed that the random forests achieved an area under the receiver operating characteristic curve of 0.9547 for the test set, which was the best compared with the other machine learning algorithms. In addition, the temperature was the most important factor in our variable importance analysis, and it showed a positive effect on dengue fever at < 30 °C but had less of an effect at > 30 °C. The AQIs were not as important as temperature, but one was selected in the process of filtering the variables and showed a certain influence on the final results.

**Conclusions:**

Our study is the first to demonstrate that AQI negatively affects dengue fever occurrence in Taiwan. The proposed prediction model can be used as an early warning system for public health to prevent dengue fever outbreaks.

**Supplementary Information:**

The online version contains supplementary material available at 10.1186/s12879-024-09220-4.

## Background

Dengue fever is caused by the dengue virus, a well-studied arboviral disease worldwide, with more than 50 million new cases reported annually [1]. There are four types of dengue viruses: DENV-1, DENV-2, DENV-3, and DENV-4. All types of viruses can cause severe diseases in humans [2]. Dengue virus transmission is a major issue in tropical and subtropical areas of the world. Because the virus is transmitted by mosquitoes, transmission intensity is limited by changes in weather and climate, such as rainfall, humidity, and temperature [3]. *Aedes aegypti* is one of the most important vectors of chikungunya, dengue, and Zika viruses. Over the past few decades, the incidence of dengue fever has continued to increase [4]. Dengue fever is primarily observed in tropical regions. The first local European dengue fever cases reported in France and Croatia occurred in 2010 [5]. Bhatt et al*.* estimated that there are 390 million dengue infections per year in the tropics, of which 96 million appear to be significant [6]. Another study also demonstrated evidence of dengue in 128 countries, with an estimated 3.97 billion population at risk [7].

### History of dengue fever outbreaks in Taiwan

In the last century (i.e., 1901, 1915, 1931, 1942–1943, 1981, 1991, 1994, 1995, and 1998), several severe dengue fever outbreaks occurred in Taiwan. Among these records, 1915, 1931, and 1942–1943 outbreaks affected the entire island [4]. In general, dengue fever is not considered a local epidemic in Taiwan, but originates in neighboring countries in Southeast Asia and is brought to Taiwan through close commercial and travel activities. Each outbreak was different, and various strains were determined to be related to those imported from Vietnam, Thailand, Indonesia, and Cambodia [1].

Between 2010 and 2015, severe outbreaks of dengue fever occurred three times in Taiwan. The first epidemic occurred in Penghu County in 2010, with a prevalence of 101 per 100,000 people. The second was in Kaohsiung City in 2014, with a prevalence rate of 540 per 100,000 population. A recent outbreak occurred in 2015 in Tainan City with a prevalence rate of 1208 per 100,000 population. The national prevalence rate in 2010 was 8 per 100,000 people (i.e., 1,888 cases), which increased to 187 per 100,000 people in 2015 (i.e., 43,832 cases). Furthermore, the prevalence rate in people aged over 50 years is higher than that in other groups [8, 9]. Currently, dengue fever outbreaks occur mainly in southern Taiwan, where *Ae. aegypti* and *Ae. albopictus* coexist, but rarely in the northern or central regions, where only *Ae. albopictus* exists. Local outbreaks are most frequent in summer and fall, less frequent in winter, and caused by a single dengue fever virus strain that disappears when the outbreak ends [10].

### Literature review of dengue fever prediction

Several methods have been proposed to analyze dengue fever. In Sakon Nakhon Province, Thailand, the incidence of dengue virus infection (DVI) is related to meteorological factors and socioeconomic covariates based on a generalized linear model. The monthly minimum temperature had a positive effect, but the 2-month cumulative rainfall had a negative effect on the DVI [11]. In Singapore, the *Ae. aegypti* breeding percentage was positively correlated with annual case counts by Spearman’s correlation coefficient across 11 years, which ranged between 0.547 and 0.737 [12]. A previous study found no relationship between air pollution index (API) levels and dengue cases in all five study zones in Selangor, Malaysia [13]. In Greater São Paulo, Brazil, statistical associations of particulate matter with an aerodynamic diameter of < 10 µm (PM_10_) and moisture were found in reported dengue fever cases. This study showed that PM_10_ had a negative effect on dengue fever [14].

Furthermore, the lag effect of variables is a key factor influencing dengue fever occurrence and has been incorporated in several studies. Chen et al*.* found that several monthly meteorological factors with lag periods of 0–4 months were positively associated with dengue fever cases in Kaohsiung, Taiwan using Spearman’s coefficient [15]. Wu et al*.* used the autoregressive integrated moving average (ARIMA) model to analyze the incidence of dengue fever and found that temperature and relative humidity with a lag effect of 2 months had significant effects on the incidence of dengue fever in Kaohsiung [16]. Chuang et al*.* analyzed weekly dengue incidence rates in a distributed lag non-linear model, which can simultaneously handle the lag effects of factors and non-linear relationships. Minimum temperature and precipitation were non-linear. The incidence rate increased when the temperature was either lower than 17 °C or higher than 23 °C. Moderate to heavy rainfall had a stronger effect than low to moderate or heavy rainfall. This might increase the risk of transmission at a lag of 10 or 20 weeks [17]. Phung et al*.* used generalized linear distributed lag models to analyze dengue cases and meteorological data of the Mekong Delta region (MDR) from 2003 to 2013, and found that the northeast MDR was a high-risk cluster. A 1 °C increase in temperature at lags of 1–4, 5–8, and 9–12 weeks increases the risk of dengue by 11, 7, and 5%, respectively. However, a 1% rise in humidity and a 1-mm increase in rainfall increased the risk by less than 1% [18]. Johansson et al*.* used Poisson regression and distributed lag models to analyze monthly dengue incidences. The temperature at lag periods of 0, 1, and 2 months and precipitation at lag periods of 1 and 2 months were positively associated with variances in monthly dengue incidence [3].

In addition to statistical methods, machine learning is a computer method that focuses on learning by experience from data [19]. Machine learning algorithms combine statistics and make predictions in a dataset by finding patterns [20]. Recently, the use of machine learning algorithms to make reliable predictions from datasets has become increasingly common [21]. Guo et al*.* utilized six machine-learning algorithms to predict the weekly incidence of dengue fever using data from 2011 to 2014 in Guangdong, China. The results showed that the support vector regression model was the best prediction model with the smallest prediction error rates for tracking the status and predicting outbreaks of dengue fever in other areas [22]. Carvajal et al*.* incorporated four models to predict dengue incidence with meteorological factors, and found that random forests with lagged meteorological factors were the best prediction models based on the minimum root mean square error and mean absolute error [23]. In Selangor, Malaysia, Salim et al*.* used machine learning techniques, including decision trees, artificial neural networks, support vector machines (SVM), and naïve Bayes, to predict dengue outbreaks. They incorporated climate variables, such as temperature, wind speed, humidity, and rainfall, into each model. The results showed that the SVM with a linear kernel had the best performance based on a test set with the highest prediction accuracy of 70% [24]. Compared to regression and ARIMA, Benedum et al*.* used dengue surveillance, population, temporal, and weather data to build models based on random forests, regression, and ARIMA to predict dengue fever counts and outbreaks in three geographic locations. They concluded that the random forests outperformed the other algorithms, with 21% and 33% fewer errors than Poisson regression and ARIMA, respectively [25].

### Specific aims of this study

Our study aimed to build a prediction model with lag times of 0–10 days, which included meteorological factors, a vector index, and air quality indices (AQIs), to predict dengue fever occurrences. Furthermore, the importance of the variables and predictors of dengue fever were analyzed. We chose the best prediction model for classification by comparing three machine learning algorithms to predict cases of dengue fever. AQIs, including PM_10_ and PM_2.5_, are reported to be important features of environmental air pollution. However, the relationship between AQIs and dengue fever remains unknown and has rarely been studied. Furthermore, the ultraviolet (UV) index is an important factor. These environmental factors were incorporated into our method to further determine their relationship with dengue fever.

## Methods

### Data source

This study attempted to identify the factors affecting the occurrence of dengue fever. From October 18, 2013, to December 31, 2015, 805 observations (days) with 57,724 infected cases were collected from open-source data from several official websites in Taiwan. Dengue cases and the vector index were collected from the Centers for Disease Control, meteorological data were obtained from the Central Weather Bureau, and the AQIs were downloaded from the Environmental Protection Administration. These datasets are publicly available and can be downloaded freely (as shown in Table 1). Meteorological variables are used to analyze and predict dengue fever cases or incidence in many countries [3, 18, 26–29]. Variable-related vectors were used in previous studies [12, 30]. In previous studies, variables related to AQIs were considered when analyzing their associations with dengue fever in other countries [13, 14]. This is the first study to incorporate Taiwan’s AQIs to identify their associations with dengue fever. In addition, to consider lag effects, 121 variables ( 11 features × and 11 lagged days) were included in our models. The main study areas included Tainan City and Kaohsiung City in southern Taiwan, which have a tropical climate. At the end of 2015, 1.7 million households and 4.7 million people lived in these areas. We chose these two cities as our study objects because 98% of all indigenous cases in Taiwan occurred in Kaohsiung and Tainan in 2015. More than half of the local dengue fever cases were recorded in Tainan City.

**Table 1:** Variables of the daily vector index, meteorological data, and air quality indices

Category	Features	Source	Description
Dengue cases	Local dengue cases	Centers for Disease Control, Taiwan^a,b^	Dengue daily confirmed cases
Vector index	BI HI LI		Dengue mosquito investigation records $\mathrm{BreteauIndex}\left(\text{BI}\right)(\%)=\frac{No.\;of\;positive\;containers\;infested}{Total\;no.\;of\;containers\;inspected}\ast100$ $\mathrm{HouseIndex}\left(\text{HI}\right)(\%)=\frac{No.\;of\;positive\;housees\;infected}{Total\;no.\;of\;houses\;inspected}\ast100$ $\mathrm{LarvaIndex}\left(\text{LI}\right)(\%)=\frac{No.\;of\;larvae}{Total\;no.\;of\;houses\;inspected}\ast100$
Meteorological data	Temperature Rainfall Relative humidity UV index Wind speed Atmospheric pressure	Central Weather Bureau, Taiwan^c^	Temperature: daily average temperature (^o^C) Rainfall: daily total rainfall (mm) Relative Humidity: daily average relative humidity (%) UV Index Wind Speed: average wind speed per hour (km/hr) Atmospheric pressure (hPa)
Air quality index	PM_10_ PM_2.5_	Environmental Protection Administration, Taiwan^d^	PM_10_(μg/m^3^): Inhalable particles with diameters which are 10 µm or smaller PM_2.5_(μg/m^3^): Inhalable particles with diameters which are 2.5 µm or smaller

*Abbreviation*: *BI* Breteau Index, *HI* House Index, *LI* Larvae Index, *UV* ultraviolet

^a^https://data.cdc.gov.tw/en/dataset/dengue-daily-determined-cases-1998

^b^https://data.cdc.gov.tw/en/dataset/dengue-mosquito-investigation-latest-12m

^c^https://opendata.cwb.gov.tw/dataset/observation?page=1

^d^https://www.epa.gov.tw/eng/5B794123D2E93D96

### Statistical analyses

SAS 9.4 (SAS Institute, Cary, NC, USA) was used to analyze baseline characteristics, and RStudio ver. The 1.2.5001 software (2009–2019 RStudio) was used to construct the prediction model. Means and standard deviations (SDs) were reported for continuous variables and compared using Student’s *t*-test to examine whether there were significant differences in the occurrence of dengue fever. For model comparison, we used the area under the receiver operating characteristic curve (AUC) as an evaluation measure to select the best prediction model.

### Machine learning algorithms

Logistic regression analyzes the relationships between a categorical variable, which is either dichotomous or multinomial, and multiple dependent variables. The regression output directly generates an effect estimate and *a p*-value [31, 32]. In 2001, Breiman proposed random forests [33], which used a bagging ensemble method to randomly create several independent and unpruned trees from a dataset. The forest generalization error converges to a limit when the number of trees generated in the model becomes larger [34]. The package, “randomForest” was developed by Breiman and Culter and is available in the R environment. XGBoost is an algorithm based on the gradient boosting decision tree [35] proposed by Chen and Guestrin. Using an additive training strategy, it combines all predictions of a group of weak learners to build a strong classifier [36]. In our study, we used the “xgboost” package in the R environment.

### Evaluation metrics

The AUC, accuracy, sensitivity, and specificity are the four measures used to evaluate prediction performance. These are defined in Eqs. (1, 2 and 3):

1 $${\text{Accuracy}}=\left({\text{TP}}+{\text{TN}}\right)/\left({\text{TP}}+{\text{TN}}+{\text{FP}}+{\text{FN}}\right)$$

2 $${\text{Sensitivity}}={\text{TP}}/\left({\text{TP}}+{\text{FN}}\right)$$

3 $${\text{Specificity}}={\text{TN}}/\left({\text{TN}}+{\text{FP}}\right)$$

Here, TP, TN, FP, and FN denote the true positives, true negatives, false positives, and false negatives, respectively.

The receiver operating characteristic (ROC) curve plot shows tradeoffs between sensitivities and specificities, in which the y-axis represents sensitivity and the x-axis denotes [1—specificity]. Models with higher sensitivity and specificity generated curves close to the upper-left corner of the ROC plot. The AUC has been used in medical research since the 1970s and is considered a standard indicator for evaluating the performance of predictive models. Previous research suggested that AUC is a better measure for comparing the performance of classifiers [37]. It summarizes a model's performance and avoids the threshold selected by the supposed subjectivity by converting a continuous probability into a binary positive–negative variable [38].

### System architecture

**Fig. 1:**
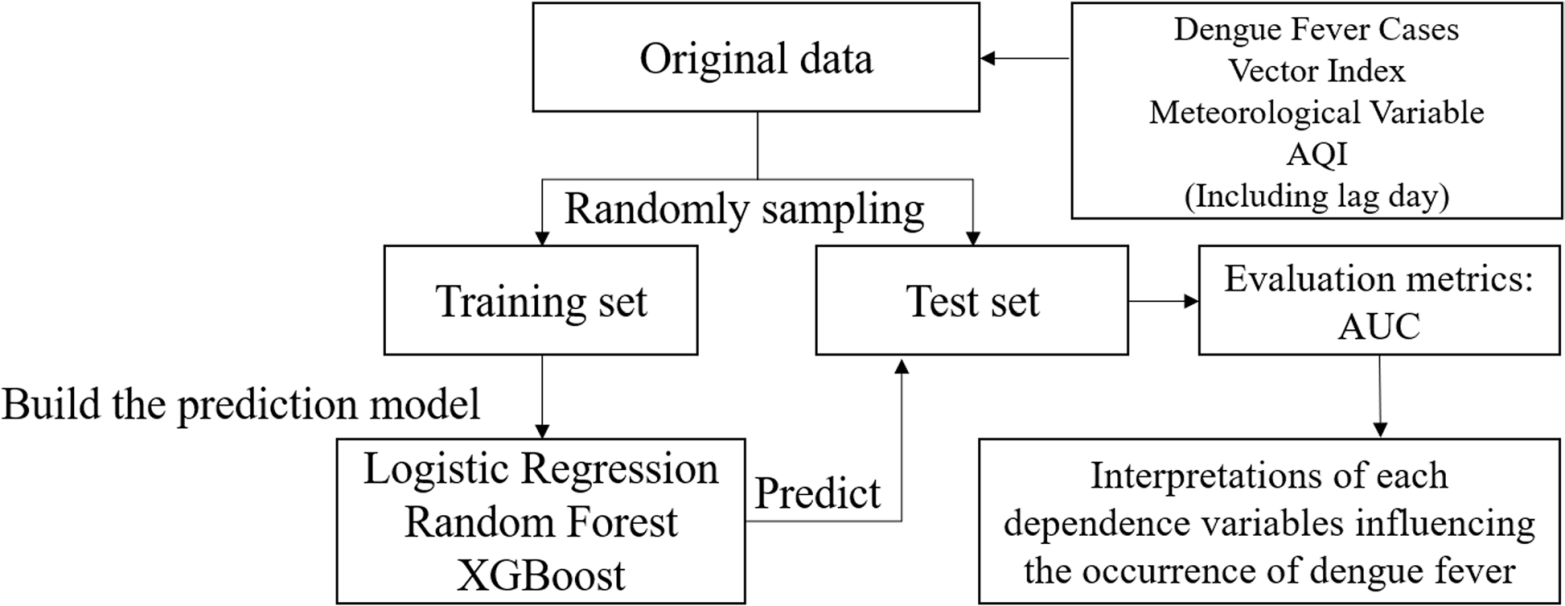
System architecture of the proposed study

The original dataset was divided into training and test sets for the development of the prediction model and validation of the predictive performance. We used three algorithms, logistic regression, random forests, and XGBoost, to build a prediction model for the occurrence of dengue fever and compared them using the AUC of the test set. Finally, we interpreted the effects of each variable on dengue fever occurrence. The workflow is illustrated in Fig. 1.

## Results

### Descriptive statistical analysis of dengue fever outbreaks

A total of 805 samples were collected, and 65.96% of the records had dengue fever. We used Student’s *t*-test for evaluation, and the means of all variables showed significant differences (as shown in Table 2) between the occurrence or non-occurrence of dengue fever. The results showed that meteorological variables and the vector index had statistically significant differences between occurrence and nonoccurrence. In AQIs (PM_10_ and PM_2.5_), the mean concentration of occurrence was significantly lower than that of non-occurrence.

**Table 2:** Descriptive statistics of variables related to the occurrence of dengue fever

	Occurrence of dengue fever				*p* value
	Yes (*n* = 531)		No (*n* = 264)		
Variable	Mean	SD	Mean	SD	
Temperature (°C)	26.22	4.06	22.83	4.20	< 0.0001***
UV index	6.68	2.60	5.96	2.03	< 0.0001***
Rainfall (mm)	9.76	36.15	3.62	13.25	0.0078**
Atmospheric pressure (hPa)	1010.50	6.03	1013.80	4.00	< 0.0001***
BI	3.72	2.89	2.45	2.00	< 0.0001***
HI	2.93	2.06	2.08	1.58	< 0.0001***
LI	41.84	54.02	28.28	26.75	< 0.0001***
PM_10_ (μg/m^3^)	60.30	30.31	73.87	29.32	< 0.0001***
PM_2.5_ (μg/m^3^)	27.38	17.67	36.64	19.66	< 0.0001***
Relative humidity (%)	74.19	6.33	71.91	6.40	< 0.0001***
Wind speed (m/s)	2.49	0.84	2.63	0.76	0.0291*

*Abbreviation*: *SD* standard deviation, *UV* ultraviolet, *BI* Breteau Index, *HI* House Index, *LI* Larvae Index

^***^p < 0.001

^**^p < 0.01

^*^p < 0.05

### Prediction of dengue fever occurrences using machine learning algorithms and lag effects

**Fig. 2:**
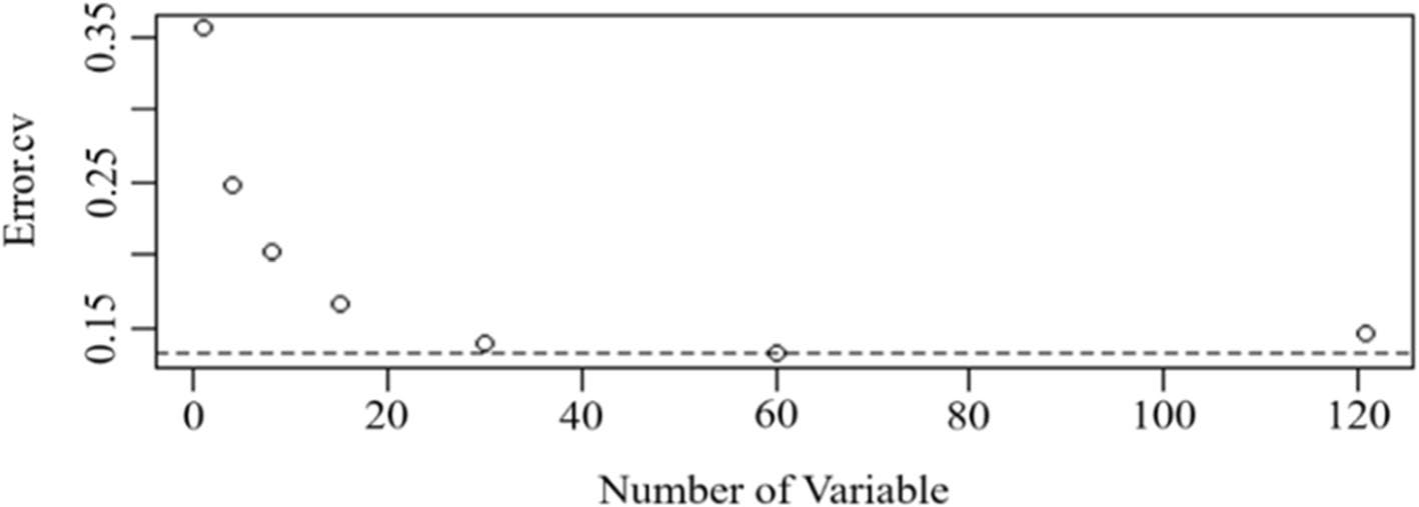
Relationships between mean square errors and a number of top variables incorporated into the model

To predict dengue fever, a random forest algorithm was first incorporated to choose the best feature set with the lowest prediction error in the feature selection. The original dataset was randomly divided into two parts: 80% as a training set with 636 observations and 20% as a test set with 159 observations. In the random forest package, the “rfcv” function was used to optimize the prediction model by examining errors under different combinations of variables. There were 121 variables (11 features × and 11 lag days) in the models. We found that the minimum error occurred for 60 variables (indicated by the dashed line in Fig. 2).

The top 60 variables generated the lowest error compared to using all variables, and the model trained on 60 variables performed slightly better than the original model using all variables in terms of both accuracy and AUC (Table 3). The new model had a higher AUC and accuracy based on fewer variables than the original model. We chose the model with the top 60 variables for comparison with other machine learning algorithms.

**Table 3:** Comparison of the random forest model with/without feature selection for the test set

	No. of variables	Acc	Sen	Spec	AUC
Random forests (all variables included)	121	0.8742	0.9688	0.7302	0.9545
Random forests (top 60 variables selected)	60	0.8994	0.9479	0.8254	0.9547

*Abbreviations*: *Acc.* Accuracy, *Sen.* Sensitivity, *Spec.* specificity, *AUC* area under the receiver operating characteristics curve

Principal component analysis (PCA) is a well-known approach for feature extraction. Kaiser criterion and scree plot were used to determine the number of components in the PCA. The Kaiser criterion drops all components with an eigenvalue < 1 [39]. The scree plot shows the top 25 components with eigenvalues > 1 (Figure S1 in Additional File 1). The top 25, 60, and all components were incorporated into the prediction model based on the random forests. The prediction model with 25 components achieved the highest AUC of 0.9512 among the three models (Table S1 in Additional File 1); however, it was lower than that of the model with the top 60 variables.

**Fig. 3:**
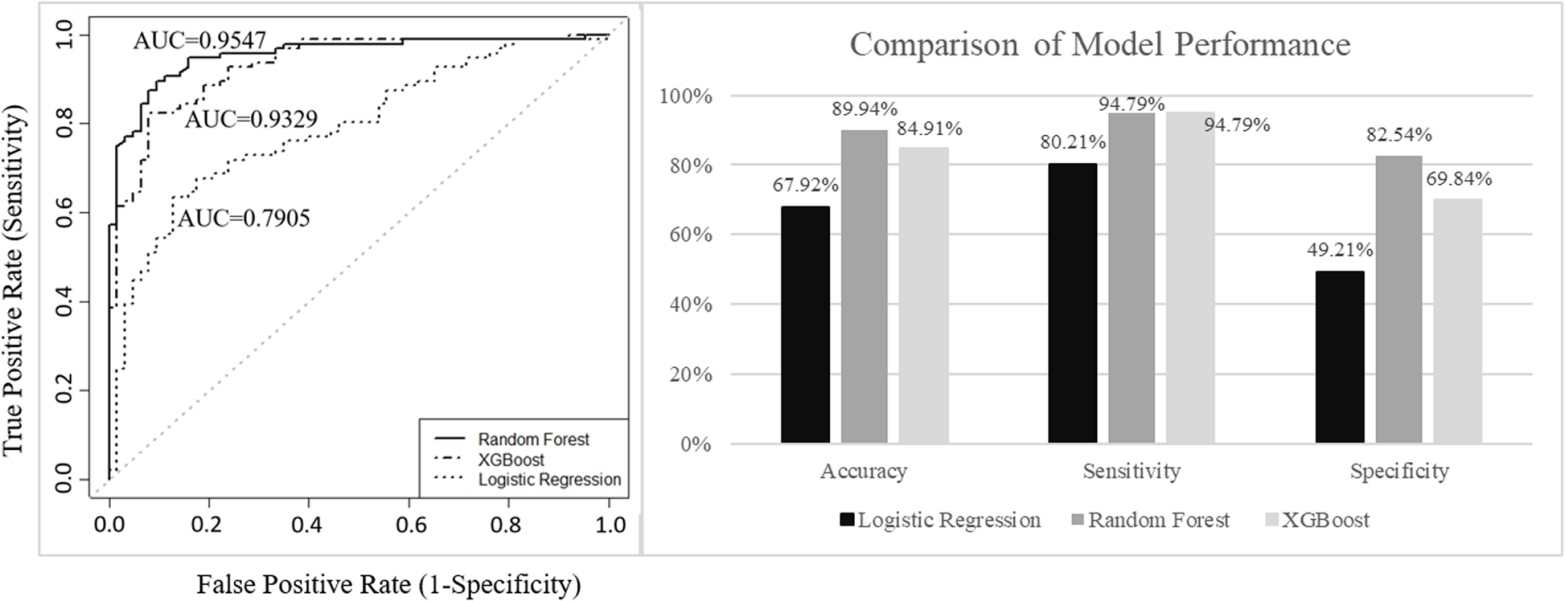
Comparison of the prediction performance based on different algorithms for the test set

For the performance comparison based on 60 selected variables, as shown in Fig. 3, the random forests outperformed the other algorithms in terms of accuracy, specificity, and AUC, except that XGBoost also performed well in terms of sensitivity. For the test set, the random forests achieved an AUC of 0.9547, compared with 0.9329 for XGBoost and 0.7905 for logistic regression. Therefore, we chose random forest as the best prediction model.

### Effects of variables on dengue fever outbreaks

**Fig. 4:**
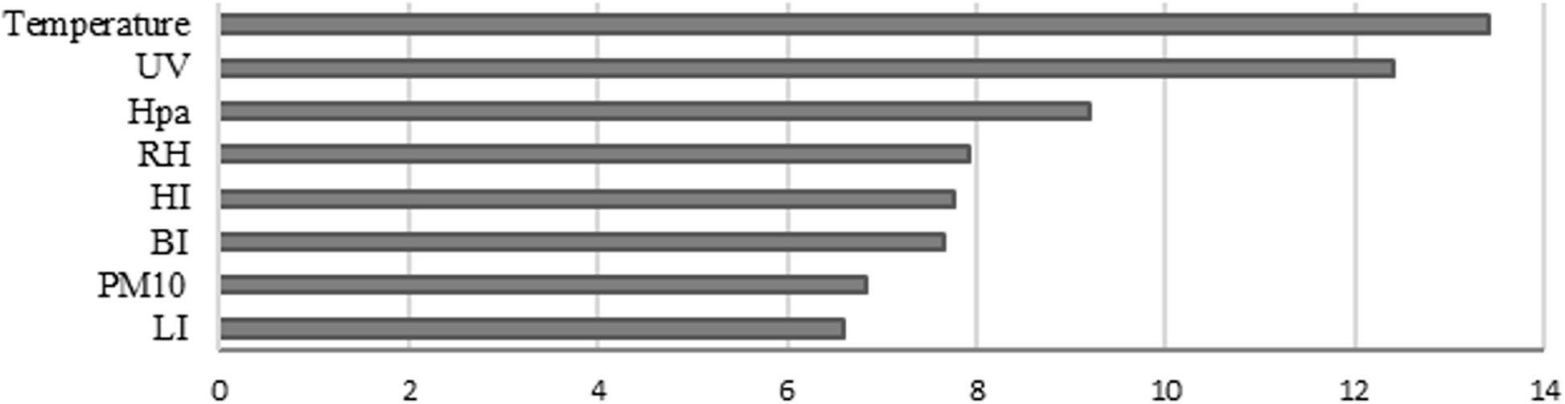
Importance of variables for predicting dengue fever occurrences

The selected variables were ranked according to the mean decrease in accuracy (MDA) estimated using our model. The MDA is an index generated by observing out-of-bag errors for a given variable. The MDA index of each variable was averaged for comparison with the others, as shown on the *x*-axis in Fig. 4. The results showed that temperature was the most important factor, and UV was the second-most important variable compared to the others. This suggests that these two variables could be considered critical factors in the occurrence of dengue fever.

**Fig. 5:**
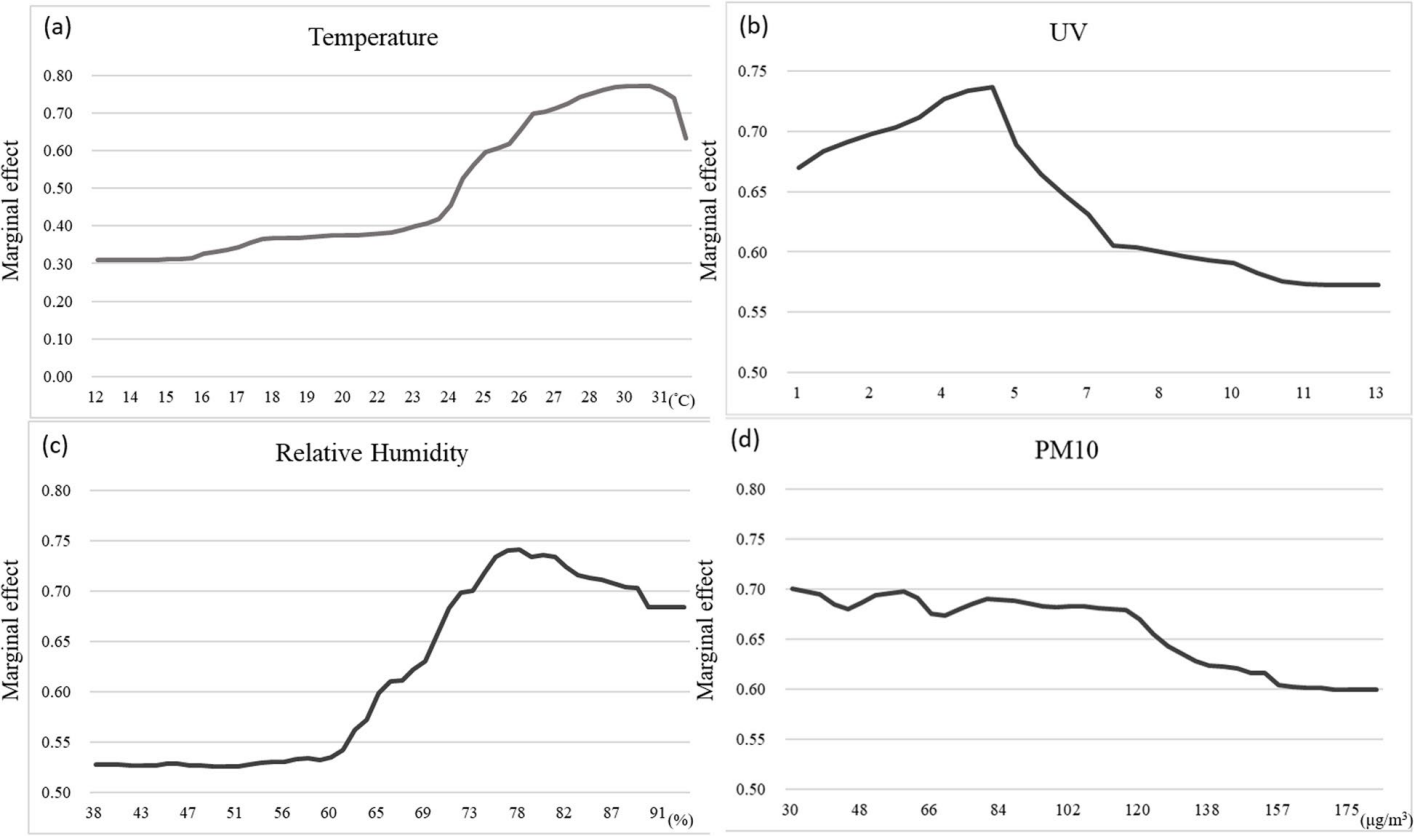
Relationships between variables and dengue fever depicted by the marginal effects from partial dependency plots

Figure 5 depicts the relationship between dengue fever occurrence and the predictors. The variables are labeled on the *x*-axis, and changes in the marginal effects of dengue fever occurrences are illustrated on the *y*-axis. The marginal effects generated by the “partial” function in the “pdp” package were averaged in the plots. Changes in the marginal effects of temperature showed a non-linear relationship, with a peak near 30 °C (Fig. 5a). Below 30 °C, the temperature has a positive effect on dengue fever occurrence. Although higher temperatures increased the probability of occurrence, an overheated environment above 30 °C decreased the occurrence. According to the World Health Organization classification, the UV index is categorized into five levels:1 and 2, low; 3–5, moderate; 6 and 7, high; 8–10, very high; and 11 + : extreme [40]. In Fig. 5b, the peak of the UV index was near 5, indicating that the marginal effect of UV increased slightly before the moderate level and began to fall afterwards. Relative humidity (RH) had a positive effect and peaked at approximately 78% (Fig. 5c). As shown in Fig. 5d, the marginal effect of PM_10_ was negative on the probability of occurrence. The figure shows that higher PM_10_ levels lead to a lower probability of dengue occurrence.

## Discussion

Our research attempted to select the best model to predict dengue fever outbreaks and investigate the relationships between predictors and dengue fever. From our experimental results, the random forests outperformed the other two algorithms in terms of AUC for predicting dengue fever outbreaks (Fig. 3, Table S2 in Additional File 1). The temperature was the most important factor influencing the occurrence of dengue fever.

In previous studies, dengue fever cases or incidence rates were used as target variables for the estimation. A time series model and generalized additive model have been used to explain predictors of dengue cases or incidence rates [16, 18, 24, 28, 41]. Compared to those studies, the random forest algorithm not only accurately predicted the occurrence of dengue fever but also provided a partial dependence plot to depict the relationships between predictors and dengue fever. The importance of a variable is generated by observing the out-of-bag error for that specific variable as other variables remain [33]. Biomedical interpretations of predictors from our results correspond well with the domain knowledge.

AUC is a good indicator for measuring the performance of prediction models. Based on our results, random forests achieved the highest AUC of 0.9547. Ghosh et al*.*. used logistic regression to predict the occurrence of dengue in Kharaghur, India. Their results showed that the AUC values for the training and testing data were 0.854 and 0.858, respectively [42]. Adde et al*.* used logistic binomial regression to predict outbreaks over the period 1991–2013 in French Guiana using climate indicators, and their best result was an AUC of 0.88 [43]. Additionally, in some previous studies, models were built to predict the epidemic years. Descloux et al*.* used an SVM explicative model to predict outbreak years in Noumea, New Caledonia, with an AUC of 0.8 [44]. Hii et al*.* used Singaporean weekly data at lag times of up to 20 weeks to develop a time-series Poisson multivariate regression to detect outbreaks (when the weekly clinical cases exceeded the epidemic threshold) in 2004–2010 and 2011. Their results showed AUCs of 0.96 in 2004–2010 and 0.98 in 2011 [45]. Compared with other studies, the prediction model based on random forests achieved a higher AUC.

Random forest achieved the highest accuracy of 89.94% in our experimental results. Nejad et al*.*. used five algorithms to construct prediction models for dengue outbreaks from 2010 to 2013. Their results showed that the Bayes network model with mean and maximum temperature, mean relative humidity, and TempeRain factor (TRF, which consists of the average of minimum temperature five weeks plus a current week and cumulative rainfall for two weeks before the current week) achieved the best accuracy of 92.35% [46]. Anno et al*.* used deep learning based on AlexNet to predict dengue fever outbreaks using a dataset of longitudinal-time sea surface temperature via eightfold cross-validation and yielded an accuracy of 100% [27]. Moreover, the highest sensitivity and specificity in our study were 0.9688 and 0.7302, respectively. Althouse et al*.* used the Singapore dataset to develop a prediction model for periods with high dengue incidence, and the results showed that the sensitivity and specificity were 0.861 and 0.765, respectively [47]. Compared with the above study, our results showed higher sensitivity but lower specificity. This result is similar to that of their study, although the outcome of interest was slight.

Temperature plays a key role in the life cycle and growth period of viruses in mosquitoes [48]. Temperature showed a non-linear relationship with and a positive effect on dengue fever occurrence, indicating that higher temperatures increased the probability of occurrence before 30 °C, which began to decrease afterwards. Colon-Gonzalez et al*.* also reported that the effect of temperature has a highly non-linear relationship with dengue incidence in their generalized additive model [28]. This demonstrates that the effect of temperature on the incidence of dengue fever is not constant. Under different conditions, the effect of temperature on dengue incidence did not increase or decrease linearly. Yang et al*.* also reported that female mosquitoes survive in a temperature range of 15–30 °C and they bite more to raise mosquito offspring [49]. Furthermore, the number of offspring peaks near 30 °C, which might be a risk for dengue outbreaks [50]. The number of mosquitoes is associated with temperature, and the probability of dengue is higher at optimal temperatures for mosquitoes.

In our study, UV had a negative effect on dengue fever outbreaks when the UV intensity exceeded a moderate level. Villena et al*.* found that the metabolic rate of *Ae. albopictus* was higher after exposure to full sun conditions on days 8 and 15 compared to the non-UV condition. This shows that UVB radiation is harmful to the larval survival of *Ae. albopictus*, owing to adverse effects on their cells [51]. Compared to *Ae. aegypti*, *Ae. albopictus* is unlikely to spread the dengue fever virus on a large scale in most situations, but speculating on its presence is easier and less threatening to the public [49]. UV radiation has a limited effect on reducing the spread of the virus, but a higher intensity has a certain effect.

Regarding the particulate matter, PM_2.5_ and PM_10_ are two important AQI measures widely used worldwide. Thiruchelvam et al*.* found no relationship between the air pollution index and dengue fever cases in five zones in Selangor, Malaysia, based on the ARIMA model [13]. Based on our research, PM_2.5_ and the related lag effect variables were removed in the process of optimizing our model. PM_10_ appeared to reduce the occurrence of disease in our study. PM_10_ was negatively correlated with dengue fever in a previous study. Although air pollution has not been confirmed in other studies, it has shown obvious influences on the life cycle of *Ae. aegypti* mosquito [14]. In our experimental results, the relationship between the larval index and PM10 was significantly negative (Tables S3, S4, and Figure S2 in Additional File 1). The AQI is not as important as temperature, but it was selected in the process of filtering the variables and had a certain influence on the final results (Tables S5 and S6 in Additional File 1).

The lifespan of mosquitoes and virus transmission is influenced by RH [26]. Phung et al*.* reported that the RH with lag times of 1 ~ 4 and 5–8 weeks in the range of 83.5 ~ 86 had a larger effect on the risk of dengue fever through a categorical distributed lag model in Vietnam [18]. In our results, an RH of approximately 77% had the greatest effect on the probability of dengue occurrence. In general, a higher RH resulted in a higher probability. The trend was similar, although the peaks were different. In Yangon, the largest city in Myanmar, Thu et al*.* reported that the rainy season (with temperatures of 23–30 °C and RH of 90%) is the peak period of survival of *Ae. aegypti* mosquitoes. High virus transmission and longer mosquito life are beneficial conditions for outbreaks [52]. According to our results, the plum rainy season in Taiwan, when temperatures and RHs are similar to those in Yangon, might be a key prevention period.

A limitation of our research is that we did not account for the effect of El Niño Southern Oscillation (ENSO). Many studies [17, 23, 26, 48, 53] have determined that ENSO is an important factor influencing climate and dengue transmission. Furthermore, transportation, clustering effect of population density, urbanization, and geography are factors affecting virus transmission, but these data are difficult to quantify accurately. These factors can be included in future prediction models. In addition, our study included 805 records from October 2013 to December 2015. In the process of collecting data, some information might not have been released or may have been missing. In the future, the completeness of the public data can be analyzed to improve our research. Furthermore, in the available public dataset, no information about the serotypes of the dengue virus was provided. The relationship between serotype and occurrence was not investigated in our study, owing to the limitations of our dataset.

## Conclusions

Prediction models built for dengue fever cases and incidence rates use statistics and machine learning models. Our study attempted to predict the occurrence of dengue fever because predictions can be used for epidemic prevention. We considered the lag times of the meteorological, vector, and AQI variables to build prediction models using machine learning tools for the probability of occurrence. In a comparison of the studies, the lag time was shorter, but the warning was immediate. Our study can also be used to predict dengue fever within a few days.

### Supplementary Information

**Additional file 1: Table S1.** Prediction performance of the random forest model with feature reduction based on a principal component analysis in different dataset partitions. **Table S2.** Comparison of predictive performance of model based on random forest, logistic regression, and XGBoost by three different split methods. **Table S3.** Pearson’s correlation coefficients between Larvae index and PM10 with different lagged days. **Table S4.** Coefficient of PM10 for predicting Larvae Index. **Table S5.** Comparison of predictive performance using features with AQI and without AQI based on random forest by 80/20 percentage split. **Table S6.** Comparison of predictive performance using features with AQI and without AQI by 10-fold cross-validation. **Figure S1.** Scree plot of the numbers of components chosen by the Kaiser Criterion. **Figure S2.** The distribution of data and regression formula.

**Additional file 2.**

## Data Availability

The complete dataset used for this study is available as a supplementary material file.

## Abbreviations

AQI: Air quality index
ARIMA: Autoregressive Integrated Moving Average
AUC: Area under the curve
DVI: Dengue virus infection
ENSO: El Niño southern oscillation
FN: False negative
FP: False positive
MDA: Mean decrease in accuracy
MDR: Mekong Delta region
PCA: Principal component analysis
ROC: Receiver operating characteristics
TN: True negative
TP: True positive
UV: Ultraviolet
XGBoost: Extreme Gradient Boosting
