# Supplementary material for: Improving dengue fever predictions in Taiwan based on feature selection and random forests
Source: BMC Infect Dis. 2024 Mar 20;24(Suppl 2):334. doi: 10.1186/s12879-024-09220-4 (PMC10953060; doi:10.1186/s12879-024-09220-4)
Supplement: Supplementary file 1 — Additional file 1: Table S1. Prediction performance of the random forest model with feature reduction based on a principal component analysis in different dataset partitions. Table S2. Comparison of predictive performance of model based on random forest, logistic regression, and XGBoost by three different split methods. Table S3. Pearson’s correlation coefficients between Larvae index and PM10 with different lagged days. Table S4. Coefficient of PM10 for predicting Larvae Index. Table S5. Comparison of predictive performance using features with AQI and without AQI based on random forest by 80/20 percentage split. Table S6. Comparison of predictive performance using features with AQI and without AQI by 10-fold cross-validation. Figure S1. Scree plot of the numbers of components chosen by the Kaiser Criterion. Figure S2. The distribution of data and regression formula. [file 12879_2024_9220_MOESM1_ESM.docx]

**Supplementary Materials**

**Table S1** Prediction performance of the random forest model with feature reduction based on a principal component analysis in different dataset partitions

| Random forests | No. of components | Acc. | Sen. | Spec. | AUC |
| --- | --- | --- | --- | --- | --- |
| All components included | 121 | 0.7044 | 0.9896 | 0.2698 | 0.9143 |
| Top 60 components selected | 60 | 0.7233 | 0.9896 | 0.3175 | 0.9415 |
| Top 25 components selected | 25 | 0.7736 | 0.9688 | 0.4762 | 0.9512 |

Abbreviation: Acc., accuracy; Sen., sensitivity; Spec., specificity; AUC, area under the curve.

Remarks: Figure S1 shows the top 25 components with an eigenvalue of >1. We chose all, the top 60, and the top 25 components to compare with the random forests including 60 variables.

**Table S2** Comparison of predictive performance of model based on random forest, logistic regression, and XGBoost by three different split methods

|  | | Random Forest | Logistic Regression | XGBoost |
| --- | --- | --- | --- | --- |
| 80/20 percentage split | Accuracy | 0.8742 | 0.6792 | 0.8491 |
|  | Sensitivity | 0.9688 | 0.8021 | 0.9479 |
|  | Specificity | 0.7302 | 0.4921 | 0.6984 |
|  | AUC | 0.9545 | 0.7905 | 0.9329 |
| 10-fold cross-validation | Accuracy | 0.8755 | 0.7522 | 0.8717 |
|  | Sensitivity | 0.9304 | 0.8268 | 0.9246 |
|  | Specificity | 0.7657 | 0.6016 | 0.7657 |
|  | AUC | 0.9215 | 0.7854 | 0.9205 |
| 80/10/10 percentage split | Accuracy | 0.8765 | 0.6790 | 0.8395 |
|  | Sensitivity | 0.9259 | 0.7593 | 0.8889 |
|  | Specificity | 0.7778 | 0.5185 | 0.7407 |
|  | AUC | 0.9180 | 0.7305 | 0.8937 |

**Table S3** Pearson’s correlation coefficients between Larvae index and PM10 with different lagged days

|  | Larvae Index | p-value |
| --- | --- | --- |
| PM10 | -0.30276 | 0.0000*** |
| PM10_lag01 | -0.30293 | 0.0000*** |
| PM10_lag02 | -0.31413 | 0.0000*** |
| PM10_lag03 | -0.31415 | 0.0000*** |
| PM10_lag04 | -0.31838 | 0.0000*** |
| PM10_lag05 | -0.30445 | 0.0000*** |
| PM10_lag06 | -0.28988 | 0.0000*** |
| PM10_lag07 | -0.28955 | 0.0000*** |
| PM10_lag08 | -0.28516 | 0.0000*** |
| PM10_lag09 | -0.26683 | 0.0000*** |
| PM10_lag10 | -0.25921 | 0.0000*** |

**Table S4** Coefficient of PM10 for predicting Larvae Index

|  | Coefficient | Standard Error | t-Stat | P-value |
| --- | --- | --- | --- | --- |
| (Intercept) | 67.54211 | 3.73467 | 18.08516 | 0.0000*** |
| PM10 | -0.46611 | 0.052105 | -8.94563 | 0.0000*** |

**Table S5** Comparison of predictive performance using features with AQI and without AQI based on random forest by 80/20 percentage split.

|  | Accuracy | Sensitivity | Specificity | AUC |
| --- | --- | --- | --- | --- |
| Random Forest (using all 121 features with AQI) | 0.8742 | 0.9688 | 0.7302 | 0.9545 |
| Random Forest (using 99 features without AQI) | 0.8679 | 0.9109 | 0.7931 | 0.9158 |

**Table S6** Comparison of predictive performance using features with AQI and without AQI by 10-fold cross-validation

|  | | Random Forest | Logistic Regression | XGBoost |
| --- | --- | --- | --- | --- |
| All 121 features with AQI | Accuracy | 0.8755 | 0.7522 | 0.8717 |
|  | Sensitivity | 0.9304 | 0.8268 | 0.9246 |
|  | Specificity | 0.7657 | 0.6016 | 0.7657 |
|  | AUC | 0.9215 | 0.7854 | 0.9205 |
| 99 features without AQI | Accuracy | 0.8692 | 0.7535 | 0.8755 |
|  | Sensitivity | 0.9247 | 0.8324 | 0.9265 |
|  | Specificity | 0.7583 | 0.5940 | 0.7725 |
|  | AUC | 0.9210 | 0.7973 | 0.9190 |


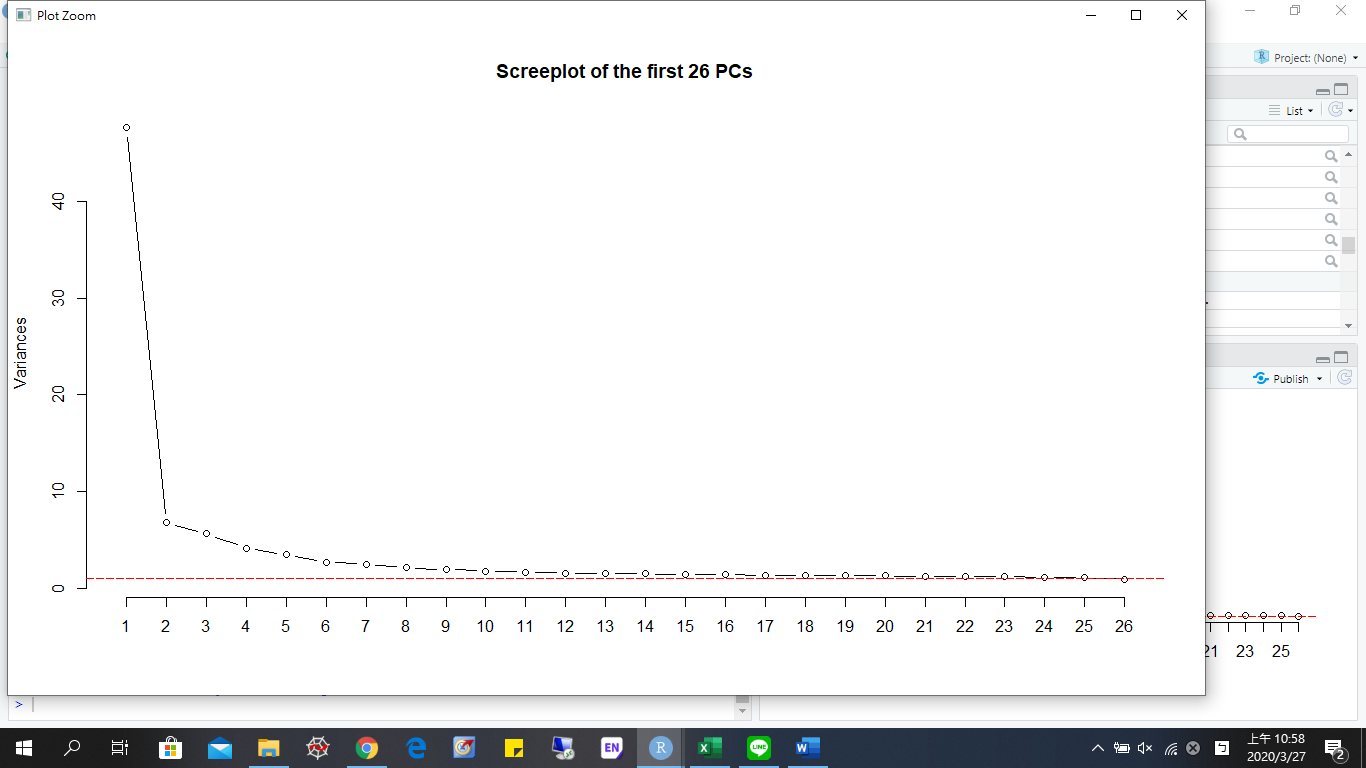


**Figure S1** Scree plot of the numbers of components chosen by the Kaiser Criterion

**Figure S2** The distribution of data and regression formula
